# Supplementary material for: Diffractive interconnects: all-optical permutation operation using diffractive networks
Source: Nanophotonics. 2022 Sep 5;12(5):905–23. doi: 10.1515/nanoph-2022-0358 (PMC11501510; doi:10.1515/nanoph-2022-0358)
Supplement: Supplementary file 1 — Supplementary Material Details [file j_nanoph-2022-0358_suppl_001.pdf]

Diffractive Interconnects: All-Optical Permutation Operation Using Diffractive Networks

Deniz Mengu[1,2,3], Yifan Zhao[1,3], Anika Tabassum[1,3], Mona Jarrahi[1,3], Aydogan Ozcan[1,2,3,4,*]

[1] Electrical and Computer Engineering Department, University of California, Los Angeles, CA, 90095, USA
[2] Bioengineering Department, University of California, Los Angeles, CA, 90095, USA
[3] California NanoSystems Institute, University of California, Los Angeles, CA, 90095, USA
[4] Department of Surgery, David Geffen School of Medicine, University of California, Los Angeles, CA, 90095, USA.
* Corresponding author: ozcan@ucla.edu

Supplementary Information (SI)

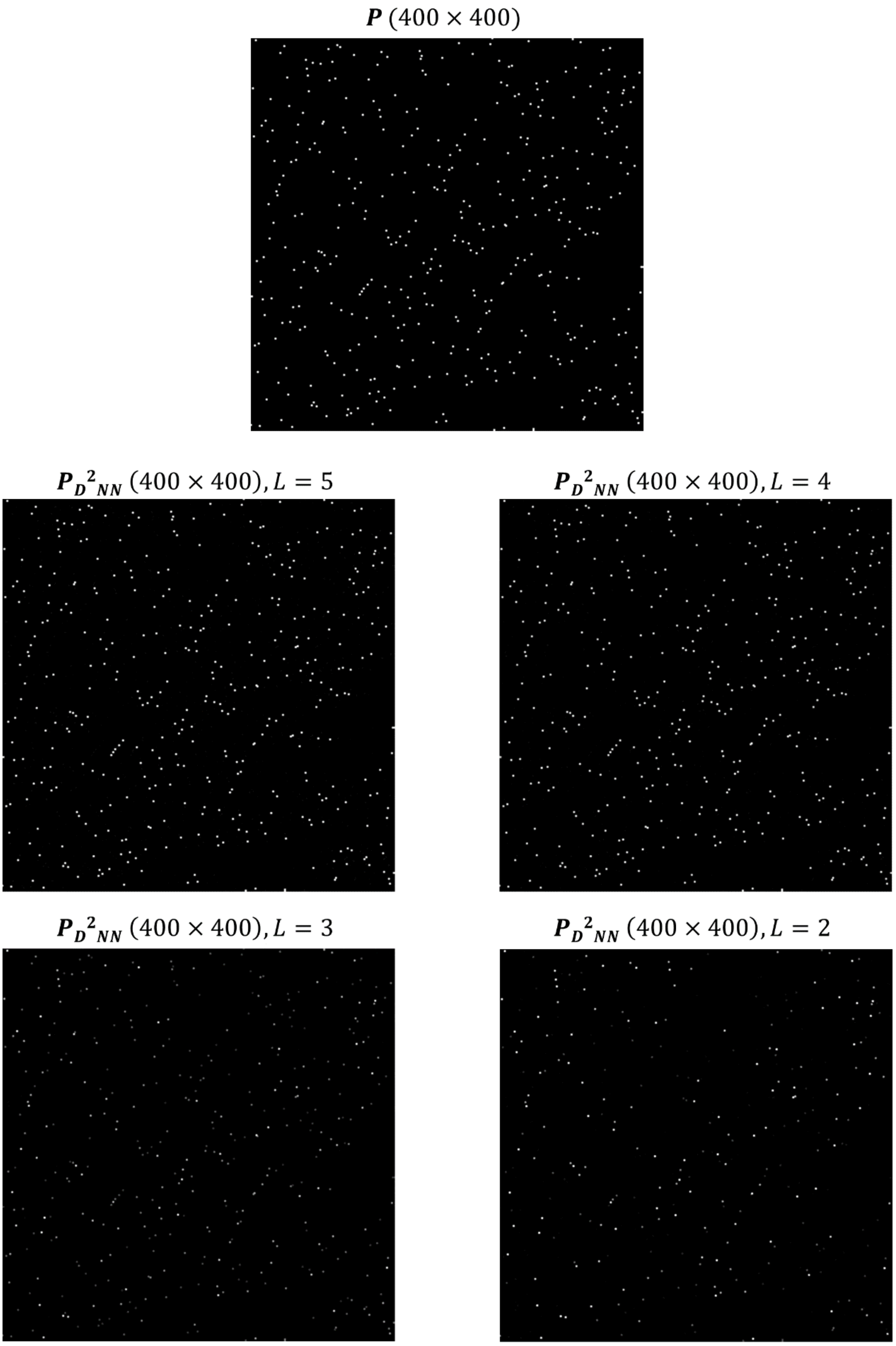

Fig. S1: Permutation matrices of size 400×400, implemented through diffractive permutation networks consisting of L=5, L=4, L=3 and L=2 diffractive layers containing 40K diffractive neurons per layer.

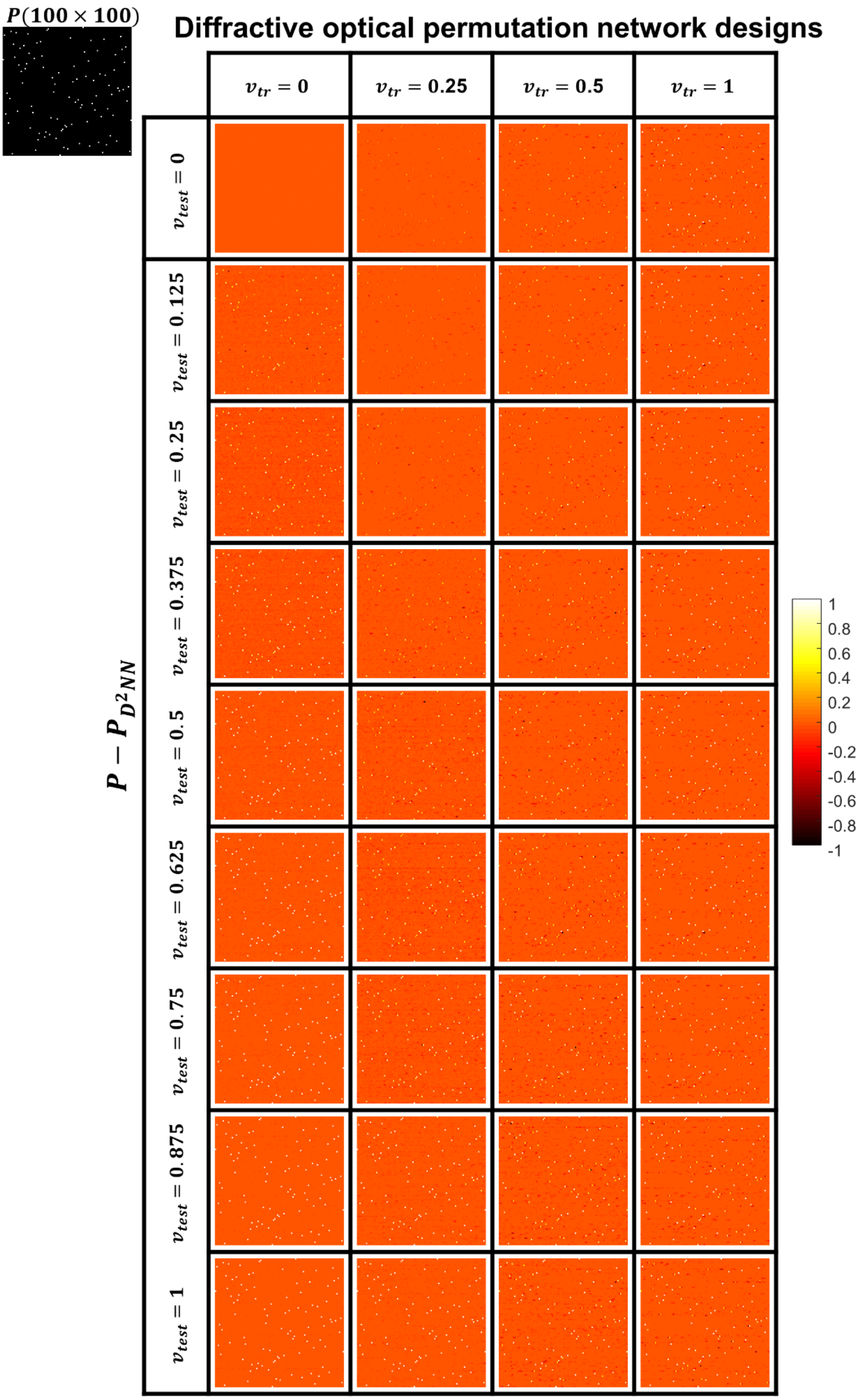

Fig. S2: Difference maps between the desired permutation matrix P and P_{D^2NN} predicted by the numerical forward model of the non-vaccinated and vaccinated diffractive permutation networks presented in Fig. 4 of the main text, under different misalignment levels.

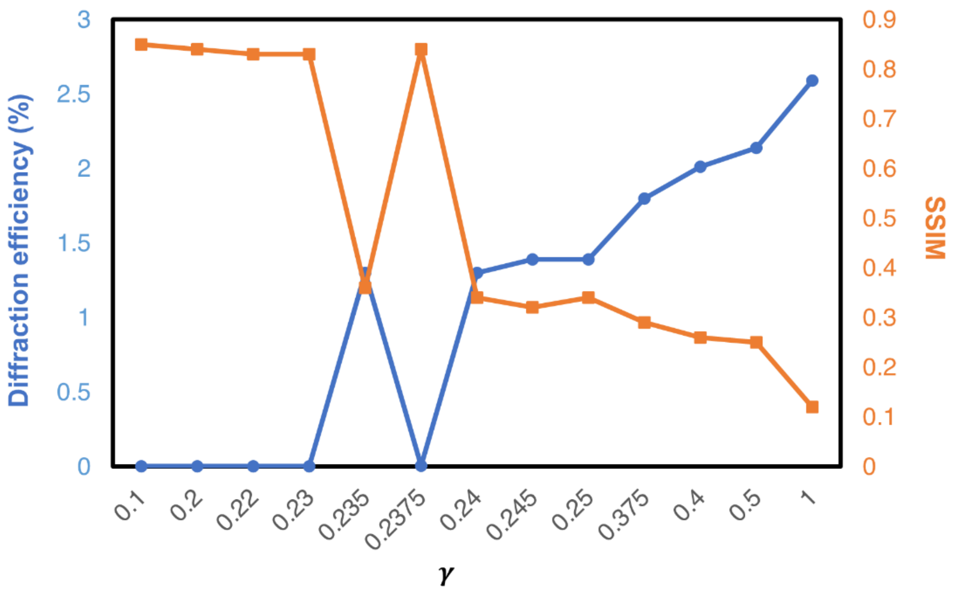

Fig. S3: The trade-off between the output diffraction efficiency and SSIM. Diffractive optical permutation networks are trained with a loss function, $\mathcal{L}' = \mathcal{L} + \gamma\mathcal{L}_e$, to optically implement a 400×400, i.e., $N_iN_o = 160\text{K}$, permutation matrix, P, based on 5 phase-only layers with 40K diffractive neurons per layer, i.e., $N = 200\text{K}$ in total. The loss function, $\mathcal{L}'$, is a linear combination of a structural loss term depicted in Eq. 5 of the main text and a diffraction efficiency promoting penalty term $\mathcal{L}_e$. The parameter γ determines the weight of the diffraction efficiency penalty (see the Methods section of the main text). The efficiency of the diffractive network model increases with γ increasing, except for the unstable region $0.235 < \gamma < 0.24$. This instability, however, is not observed when $N \gg N_iN_o$; for instance, in our experimentally validated diffractive permutation network design, $\gamma = 0.15$ provides 2.45% output diffraction efficiency despite the fact that 89.37% of the input optical power is lost due to material absorption.
